# In the picture: disulfide-poor conopeptides, a class of pharmacologically interesting compounds

**DOI:** 10.1186/s40409-016-0083-6

**Published:** 2016-11-07

**Authors:** Eline K. M. Lebbe, Jan Tytgat

**Affiliations:** Toxicology and Pharmacology, KU Leuven, O&N2, Box 922, Herestraat 49, 3000 Leuven, Belgium

**Keywords:** Cone snail, Conopressin, Contryphan, Conantokin, Contulakin, Conorfamid, Conophan, Conomap, Conomarphin, Conolysin, ConoGAY, ConoCAP, Cono-NPY

## Abstract

During evolution, nature has embraced different strategies for species to survive. One strategy, applied by predators as diverse as snakes, scorpions, sea anemones and cone snails, is using venom to immobilize or kill a prey. This venom offers a unique and extensive source of chemical diversity as it is driven by the evolutionary pressure to improve prey capture and/or to protect their species. Cone snail venom is an example of the remarkable diversity in pharmacologically active small peptides that venoms can consist of. These venom peptides, called conopeptides, are classified into two main groups based on the number of cysteine residues, namely disulfide-rich and disulfide-poor conopeptides. Since disulfide-poor conotoxins are minor components of this venom cocktail, the number of identified peptides and the characterization of these peptides is far outclassed by its cysteine-rich equivalents. This review provides an overview of 12 families of disulfide-poor peptides identified to date as well as the state of affairs.

## Background

Venom peptides offer a unique and extensive source of chemical diversity as they are driven by evolutionary pressure to improve prey capture and/or protect their species. Among the venomous species, cone snails can be seen as an interesting source of peptide-based therapeutics since they produce venoms that contain a structural and functional diversity of neurotoxins.

### Cone snails as a natural source of bioactive molecules

*Conus* is a genus of sea snails that comprises more than 700 different species, appearing naturally in tropical and subtropical marine waters [1]. The venom used by cone snails is a very complex mixture consisting of more than 100 to 1000 different bioactive components and its composition differs from species to species. Therefore, it is estimated that more than 700,000 different pharmacologically active components can be addressed to this genus [2, 3]. To date, only 0.2 % out of this remarkable number of components has been functionally and structurally investigated. Nevertheless, the consideration of *Conus* venoms as gold mines for the discovery of new therapeutics is validated by the knowledge that, out of the limited number of studied conopeptides, at least nine peptides have reached human clinical trials, and one was approved as analgesic in 2004 [4–6].

Conopeptides are classified into two main groups based on the number of cysteine bonds, namely disulfide-rich and disulfide-poor conopeptides. The term “conopeptides” is often associated with cysteine-poor peptides, while cysteine-rich peptides are more usually recognized as conotoxins. Conopeptides and conotoxins are both deployed as toxic components and are likely to act in synergy to promote a very potent effect [7]. The focus of this review is disulfide-poor peptides, containing none or only one disulfide bond. This group is subdivided into contulakins (interacting with neurotensin receptors), conantokins (interacting with N-methyl-D-aspartate receptors), conorfamides (interacting with RFamide receptor), conolysins (interacting with cellular membranes), conopressins (interacting with vasopressin receptors), contryphans (interacting with Ca_V_ or K_V_ channels), conophans (target unknown), conomarphins (target unknown), conomaps (target unknown), conoCAPs (target unknown), cono-NPYs (target unknown) and conoGAYs (target unknown). Each of these subgroups will be discussed in detail in the next sections.

According to Puillandre et al. [3], the so-called cysteine-poor conopeptides seem to be closely related to cysteine-rich conopeptides according to their signal sequences, suggesting that a distinction based on cysteine content and configuration is not phylogenetically relevant and does not reflect the evolutionary history of conopeptides. As such, some of them share highly similar signals with known superfamilies (such as contryphan with O2 and conomarphin with M). Two other superfamilies, B and C for conantokins and contulakins respectively, were identified [3]. In the next sections, we will first describe conopeptides that have a single disulfide bond (conopressins, contryphans, conoCAPs and conoGAYs). Subsequently, conopeptides without cysteine residues are described. Finally, two recently discovered hormone-like peptides are discussed.

## Conopressins

### General features of conopressins

The first conopressins, conopressin-G and conopressin-S, were described in 1987 by Cruz et al. [8]. The authors observed a characteristic “scratching” effect upon intracerebral injection in mice, similar to that elicited by vertebrate neurohypophyseal hormones. Conopressin-S is isolated from *Conus striatus*, whereas conopressin-G is purified from *Conus geographus* venom (Table 1) [8]. Later, conopressin-G was also found in *Conus imperialis* venom [9] as well as in tissue extracts of the nonvenomous snails *Lymnaea stagnalis* and *Aplysia californica* and the leech *Erpobdella octoculata* [10–12]. γ-conopressin-vil is the only conopressin to date including a γ-carboxyglutamate in its amino acid structure. This γ-carboxyglutamate residue mediates calcium binding which results in structural changes of γ-conopressin-vil [13].

**Table 1:** Characteristics of conopressins. Their amino acid sequences, respective species, target, clinical potential and references are indicated

Peptide	Amino acid sequence	Species	Target	Clinical potential	Source
Lys-conopressin-G	CFIRNCPKG^a^	*C. geographus*	vasopressin R	cardiovascular/mood	[8, 9]
Arg-conopressin-S	CIIRNCPRG^a^	*C. striatus*	V_1b_R, OTR > V_1a_R		[8]
γ-Conopressin-vil	CLIQDCPγG^a^	*C. villepinii*	?		[13]
Conopressin-T	CYIQNCLRV^a^	*C. tulipa*	V_1a_R, OTR		[17]
Conopressin-Tx	CFIRNCOP	*C. textile*	?		[68]

^a^N-terminal amidation; γ: gamma-carboxyglutamate; O: hydoxyproline; OTR: oxytocin-receptor; V_1a_R: vascular vasopressin receptor, V_1b_R: pituitary vasopressin receptor

### Vasopressin/oxytocin receptors and pharmacological properties of conopressin T

Vasopressin (AVP, amino acid sequence CYFQNCPRG^*^) and oxytocin (OT, amino acid sequence CYIQNCPLG^*^) were originally identified as neurohypophysial hormones in mammals with cyclic structures. In humans, the actions of AVP are mediated by stimulation of tissue-specific G-protein-coupled receptors (GPCRs) called vasopressin receptors (vascular V_1a_R, pituitary V_1b_R, and renal V_2_R), whereas OT acts via one OT receptor (OTR). In mammals, vasopressin and oxytocin are multifunctional hormones that fulfill important functions in osmoregulation and endocrine control [14]. Peripherally, they regulate water balance [15], the control of blood pressure, and contraction of uterine smooth muscle and mammary gland myoepithelium [14]. Centrally, these peptides affect levels of aggression, depression and bonding [16]. However, vasopressin/oxytocin-related peptides are not restricted to vertebrates since endogenous analogues have been reported in non-mammalian vertebrates, annelids, mollusks and insects, suggesting an old lineage for these peptides [17].

Conopressin-T, discovered by Dutertre et al. [17], belongs to the vasopressin-like peptide family and displays high sequence homology to the mammalian hormone oxytocin and to vasotocin (CYIQNCPRG^*^), the teleost fish equivalent of vasopressin. Pharmacological characterizations of conopressin-T across human receptors revealed that it is a selective V_1a_ antagonist, with partial agonist activity at the OT receptor and no detectable activity at V_1b_ and V_2_ receptors [17]. Most peptides of the conopressin family contain two conserved amino acids within the exocyclic peptide (Pro^7^ and Gly^9^), which are replaced with Leu^7^ and Val^9^ in conopressin-T. Both amino acid positions seem to have important implications in the binding of conopressin to its target. Whereas conopressin-T only binds to OT and V_1a_ receptors, an L7P analogue had increased affinity for the V_1a_ receptor and weak V_2_ receptor binding. Looking at the NMR structures of both conopressin-T and the L7P analogue, a difference in the orientation of the exocyclic tripeptide was observed, explaining the selectivity profile switch. Moreover, the authors observed that when Gly^9^ was replaced with Val^9^ in OT and AVP, the peptides’ activity was converted from agonist to antagonist, revealing this position as an agonist/antagonist switch at the V_1a_ receptor [17].

### Biological role in the venom

The existence of homologues of the vasopressin/oxytocin family of peptides in cone snail venom is thought to be a result of evolution from pre-existing endogenous snail peptides [8, 18]. As such, it is suggested that these peptides have been continuously used in nervous systems but have evolved opportunistically for specialized functions [8]. An example is conopressin-T. This peptide has seven out of nine residues identical to teleost fish vasotocin (AVT), including the disulfide containing ring. Since *C. tulipa* preys on fish, it is very likely that conopressin-T specifically targets teleost AVT receptors [17]. The presence of vasopressin/oxytocin-related peptides in invertebrates is thought to be a duplication of an ancestral gene that occurred before the divergence of vertebrates and invertebrates over 600 million years ago [19].

## Contryphans

### General features of contryphans

The first *D*-amino acid containing conotoxin, contryphan-R, was purified from *Conus radiatus* [20]. Most of the contryphans share the conserved sequence motif, CO(*D*-W or *D*-L)XPWC^*^, that includes a tryptophan or leucine in the *D*-configuration, a disulfide bond between the two cysteines, C-terminal amidation and in most cases hydroxylation of a proline residue preceding the *D*-Trp residue [21]. The N-terminal Cys-Pro/Hyp peptide bond exhibits *cis*-*trans* isomerization, but the more abundant *cis* isomer is believed to be the functionally relevant conformer. The contryphan motif is a robust structural scaffold that maintains the backbone structure independent of the amino acid that is substituted at residue X. This residue is Gln in contryphan-Sm, Glu in contryphan-R, and Lys in contryphan-Vn (Table 2) [22]. The bromination of Trp^7^ in contryphan-R yields *L*-6-bromotryptophan [21]. Mediated by their disulfide bond, contryphans are cyclic peptides and thus provide an interesting scaffold for molecular design [23].

**Table 2:** Characteristics of contryphans. Their amino acid sequences, respective species, observed activity and references are indicated

Peptide	Amino acid sequence	Species	Activity	Source
Contryphan-R	GCOWEPWC^a^	*C. radiatus*	“Stiff-tail” syndrome in mice	[20]
[des-Gly1]contryphan	COWEPWC^a^	*C. radiatus*	“Stiff-tail” syndrome in mice	[20]
Bromocontryphan-R	GCOWEPW^+^C^a^	*C. radiatus*	“Stiff-tail” syndrome in mice	[21]
Contryphan-Sm	GCOWQPWC^a^	*C. stercusmuscarum*	“Stiff-tail” syndrome in Webster mice	[69]
Contryphan-P	GCOWDPWC^a^	*C. purpurascens*	“Stiff-tail” syndrome in Swiss Webster mice/inhibits HVA Ca^2+^ channels	[69]
Leu-contryphan-P	GCVLLPWC	*C. purpurascens*	Body tremor and mucous secretion in fish	[70]
Contryphan-Tx	GCOWQPYC^a^	*C. textile*	“Stiff-tail” syndrome and paralysis of extremities in Swiss Webster mice	[71]
Leu-contryphan-Tx	CVLYPWC	*C. textile*	Folding and drooping of dorsal fins and passivity in Siamese fighting fish	[71]
Contryphan-Vn	GDCPWKPWC^a^	*C. ventricosus*	? (VGPC, Ca^2+^ dependent)	[24, 72]
Glacontryphan-M	NγSγCPWHPWC^a^	*C. marmoreus*	Inhibits L-type VGCC	[22, 73]
Am975	GCPWDPWC^a^	*C. amadis*	Inhibits HVA Ca^2+^ channels	[25]
Lo959	GCOWDPWC^a^	*C. loroisii*	Activates HVA Ca^2+^ channels	[25]
Contryphan-fia	GCODWQPWC	*C. figulinus*	?	[74]
[W8S]contryphan-Vn	GDCPWKPSC^a^	*C. ventricosus*	?	[75]
Contryphan-fib	GCOWMPWC^a^	*C. figulinus*	?	[76]

^a^C-terminal amidation; W^+^: 6-L-bromotryptophan; O: hydroxyproline; W: *D*-tryptophan; L: *D*-leucine; γ: gamma-carboxyglutamate; VGPC: voltage-gated potassium channel, VGCC: voltage-gated calcium channel, HVA: high voltage-activated

**Fig. 1:**
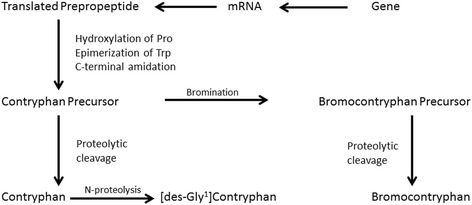
Pathway of contryphan formation. It is presumed that bromination precedes the final proteolytic cleavage. The presence of two non-brominated forms, contryphan and [des-Gly^1^]contryphan, in native venom may be due to incomplete post-translational processing with the [des-Gly^1^]analog arising from a non-physiological post-mortem proteolysis. Reprinted with permission from Jimenez et al. [21]. Copyright by the American Chemical Society (1997)

With the discovery of bromocontryphan and the identification of an encoding cDNA for a precursor, Jimenez et al. [21] suggested that three related peptides are generated in *C. radiates* venom ducts through the pathway in Fig. 1 [21].

### Pharmacological properties of contryphans

**Fig. 2:**
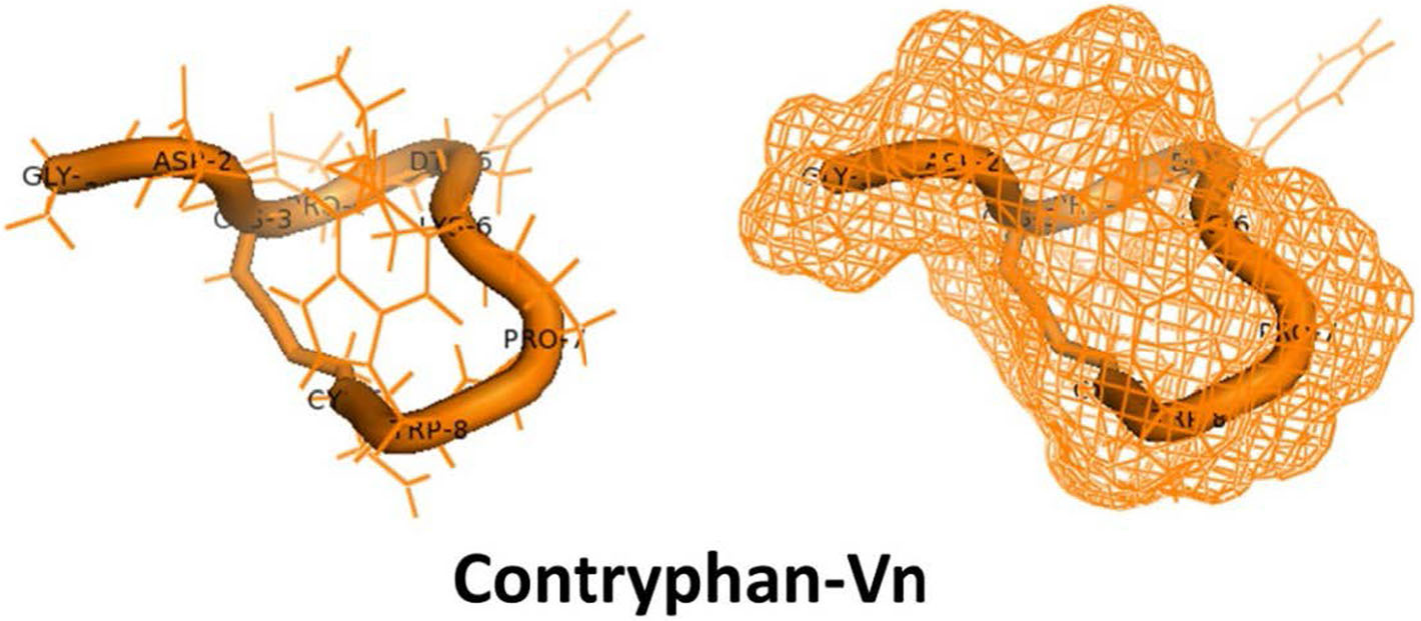
Three-dimensional representation of contryphan-Vn indicating amino acids and disulfide bond (*left*) and mesh structure (*right*). Figures were created with Pymol [82] (PDB 1N3V)

Contryphan-Vn (Fig. 2) is a *D*-Trp-containing contryphan isolated from *Conus ventricosus* that has a positively charged Lys residue within the intercysteine loop [24]. This intercysteine basic residue, Lys^6^ participates in a strong salt bridge with Asp^2^. By performing electrophysiological experiments on insect neurosecretory cells as well as in rat chromaffin cells, Massilia et al. [24] identified contryphan-Vn as a weak voltage-gated and Ca^2+^-dependent K^+^ channel modulator (> 10 μM). However, despite this weak response, it is unlikely that potassium channels are the real targets of contryphan-Vn. Also, binding experiments carried out on membrane preparations of transformed HEK 293 cells overexpressing human VGPC, K_V_1.1 and K_V_1.2 did not evidence a competition of contryphan-Vn for the BgK (sea anemone neurotoxin) binding site [24].

Glacontryphan-M is a contryphan purified from *Conus marmoreus* venom that contains Gla (γ-carboxyglutamic acid) residues [22]. These Gla residues hold a malonate-like side chain which can chelate divalent metal ions. As such, the binding of calcium ions to glacontryphan-M induces perturbations of the N-terminal residues Gla^2^, Ser^3^and Gla^4^, and the Cys^11^-Cys^5^-Pro^6^ region of the intercysteine loop, resulting in an increased exposure and slight reorientation of the stacked aromatic rings of His^8^ and *D*-Trp^7^ relative to the positioning of Trp^10^.

### Biological role in the venom

The biological activity of contryphans is that of causing a “stiff-tail” syndrome in mice when injected intracranially or triggering body tremor and mucous secretion when injected intramuscularly into fish [22]. At higher doses, contryphan-R induces more generalized excitatory effects in mice such as barrel rolling and seizures [20]. The first identification of a functional target for a contryphan was for contryphan-Vn from *Conus ventricosus,* which affects both voltage-gated and Ca^2+^-dependent potassium channels [22]. Later, glacontryphan-M was identified as a calcium-dependent antagonist of L-type voltage-gated Ca^2+^ channels (VGCC) expressed in mouse pancreatic β-cells.

The structural investigation of glacontryphan-M suggests that the function of Gla in VGCC blockage by glacontryphan-M is one of the following [22]:

1. To modify the structure of glacontryphan-M so that functional determinants interact with the channel.
2. To enable glacontryphan-M to bind to the membrane surface. The Gla residues facilitate the interaction with specific calcium channel sites via bridging of calcium and membrane components.
3. To ensure a localized enrichment of calcium ions at the channel opening which facilitates a change in the structure of the channel. This allows glacontryphan-M to bind to an antagonist-binding site with higher affinity.

Other contryphans targeting Ca^2+^ channels are Lo959 an Am975 (Table 2), identified in the venom glands of *Conus loroisii* and *Conus amadis*, respectively. Both Lo959 and Am975, which differ only in one posttranslational modified residue, target high voltage-activated Ca^2+^ channels. Intriguingly, while Lo959 increases the Ca^2+^ current, Am975 causes inhibition of VGCC [25].

## ConoCAPs

### General features of conoCAPs

ConoCAPs or cardioactive peptides from cone snails are described by Möller et al. [26]. The conoCAPs (a, b, c, Table 3) reported in this work have up to 78 % of sequence homology with crustacean cardioactive peptides (CCAP, amino acid sequence PFCNAFTGC^*^). The discovery of conoCAPs in cone snail venom emphasizes the significance of their gene plasticity to have mutations as an adaptive evolution in terms of structure, cellular site of expression, and physiological function.

**Table 3:** Characteristics of conoCAPs. Their amino acid sequences, respective species and references are indicated

Peptide	Amino acid sequence	Species	Target	Source
ConoCAP-a	PFCNSFGCYN^a^	*C. villepinii*	?	[26]
ConoCAP-b	VFCNGFTGCG^a^	*C. villepinii*	?	[26]
ConoCAP-c	LFCNGYGGCRG^a^	*C. villepinii*	?	[26]

^a^C-terminal amidation

Contrary to CCAP, conoCAP-a decreases heart rate (HR) and blood pressure in rats. These effects are in accordance with the reduced systolic calcium amplitude and contraction recorded in single ventricular myocytes from rat hearts. Moreover, analogs of conoCAP-a were used to determine structure-function relationships. The reduction in HR was lost when the Cys residues were substituted by Ala or when they were S-methylated. On the other hand, less significant changes were observed when intracystine amino acids were replaced by Ala [26].

### Pharmacological properties of conoCAPs

When conoCAP-a was applied on rat cardiac myocytes, the amplitude of systolic [Ca^2+^]_i_ and contractile activity gradually decreased. Therefore, it was expected that conoCAP-a would affect L-type Ca^2+^ channel (LTCC) current since systolic Ca^2+^ transient in cardiac muscle is triggered by Ca^2+^ entry via these channels. However, voltage-clamp experiments measuring LTCC showed that conoCAP-a had no effect on LTCC current, ruling out LTCC as a target of the peptide. Likewise, conoCAP-a did not affect other cell membrane channels and membrane receptors involved in the cardiovascular physiology. Further studies are needed to indicate the specific target of conoCAP-a.

## ConoGAYs

### General features of conoGAYs

ConoGAYs are a new class of disulfide-poor conotoxins with one disulfide bond, recently found in the venom gland of *Conus australis*. The name conoGAY originates from the first three amino acids of the peptide AusB (Table 4). Contrary to most disulfide-poor conotoxins, no post-translational modifications were observed in AusB. The authors investigated the effect of conoGAY-AusB on a large panel of ion channels. As such, the peptide was electrophysiologically screened against a panel of Na_V_s, K_V_s and nAChRs as expressed heterologously in *Xenopus laevis* oocytes. In addition, a broad screening was performed against a collection of microorganisms (29 gram-negative bacteria, ten gram-positive bacteria and two yeast strains) [84]. Unfortunately, the real target of this peptide remains to be revealed.

**Table 4:** Characteristics of conoGAY-AusB. Its amino acid sequence, respective species and reference are indicated

Peptide	Amino acid sequence	Species	Target	Source
ConoGAY-AusB	GAYFDGFDVPCVPRRDDC	*C. australis*	?	Unpublished data, [84]

## Conantokins

### General features of conantokins

Conantokins are a class of conopeptides (17–27 amino acids) without cysteine residues that selectively influence NMDA (N-methyl-D-aspartate) receptors (Table 5) [4, 27]. Conantokin-G (Con-G) from *Conus geographus* was introduced in 1984 as “the sleeper peptide”, because it induces a sleep-like state in mice when injected intracerebrally [28]. Similarly, conantokin-T from *Conus tulipa*, discovered by Haack et al. [29], induces sleep-like symptoms in young mice (10–13 days old); however, these manifestations are of smaller duration than those produced by Con-G. On account of these sleep-like symptoms, the conopeptides were named after the Philippino word for sleepy, antokin, resulting in “conantokin” [30]. Curiously, in mice older than three weeks, the same peptides induce hyperactive behavior which is more dramatic for Con-T than for Con-G [29, 31]. Later it was shown that both Con-G and Con-T interact with NMDA receptors in an antagonistic way [30, 32], produced by a competitive antagonism at the glutamate binding site of the NMDA receptor [27].

**Table 5:** Characteristics of conantokins. Their amino acid sequences, respective species, target, clinical potential and references are indicated

Peptide	Amino acid sequence	Species	Target	Clinical potential	Source
Conantokin-G	GEyyLQyNQyLIRyKSN^a^	*C. geographus*	NR2B > NR2C, NR2D	pain/epilepsy	[28, 77]
Con-T	GEyyYQKMLyNLRyAEVKKNA^a^	*C. tulipa*	NR2B, NR2A		[29]
Con-R	GEyyVAKMAAyLARyNIAKGCKVNCYP^a^	*C. radiatus*	NR2B, NR2A		[35]
Con-L	GEyyVAKMAAyLARyDAVN^a^	*C. lynceus*	?		[78]
Con-Ca2	GYγγRγIAγTVRγLEEA	*C. caracteristicus*	?		[79]
Con-Gm	GAKyRNNAyAVRyRLEEI	*C. gloriamaris*	?		[79]
Con-Qu	GYγγRγVAγTVRγLDAA	*C. quercinus*	?		[79]
Con-Oc	GEγγRKAMAγLEAKKAQγALKA	*C. orchroleucus*	?		[79]
Con-Pr1	GEDγYAγGIRγYQLIHGKI	*C. parius*	NR2B, NR2D		[80]
Con-Pr2	DEOγYAγAIRγYQLLKYGKI	*C. parius*	NR2B < NR2D		[80]
Con-Pr3	GEOγVAKWAγGLRγKAASN^a^	*C. parius*	NR2B, NR2D		[80]
Con-E	GEγγHSKYQγCLRγIRVNNVQQγC	*C. ermineus*	?		[77]
Con-P	GEγγHSKYQγCLRγIRVNKVQQγC	*C. purpurascens*	NR2B, NR2A		[77]
Con-Br	GDγγYSKFIγRERγAGRLDLSKFP	*C. sulcatus (brettinghami)*	NR2B, NR2D, NR2A < NR2C		[81]

^a^N-terminal amidation; Gla: γ-carboxyglutamic acid; NR2: NMDA receptor

**Fig. 3:**
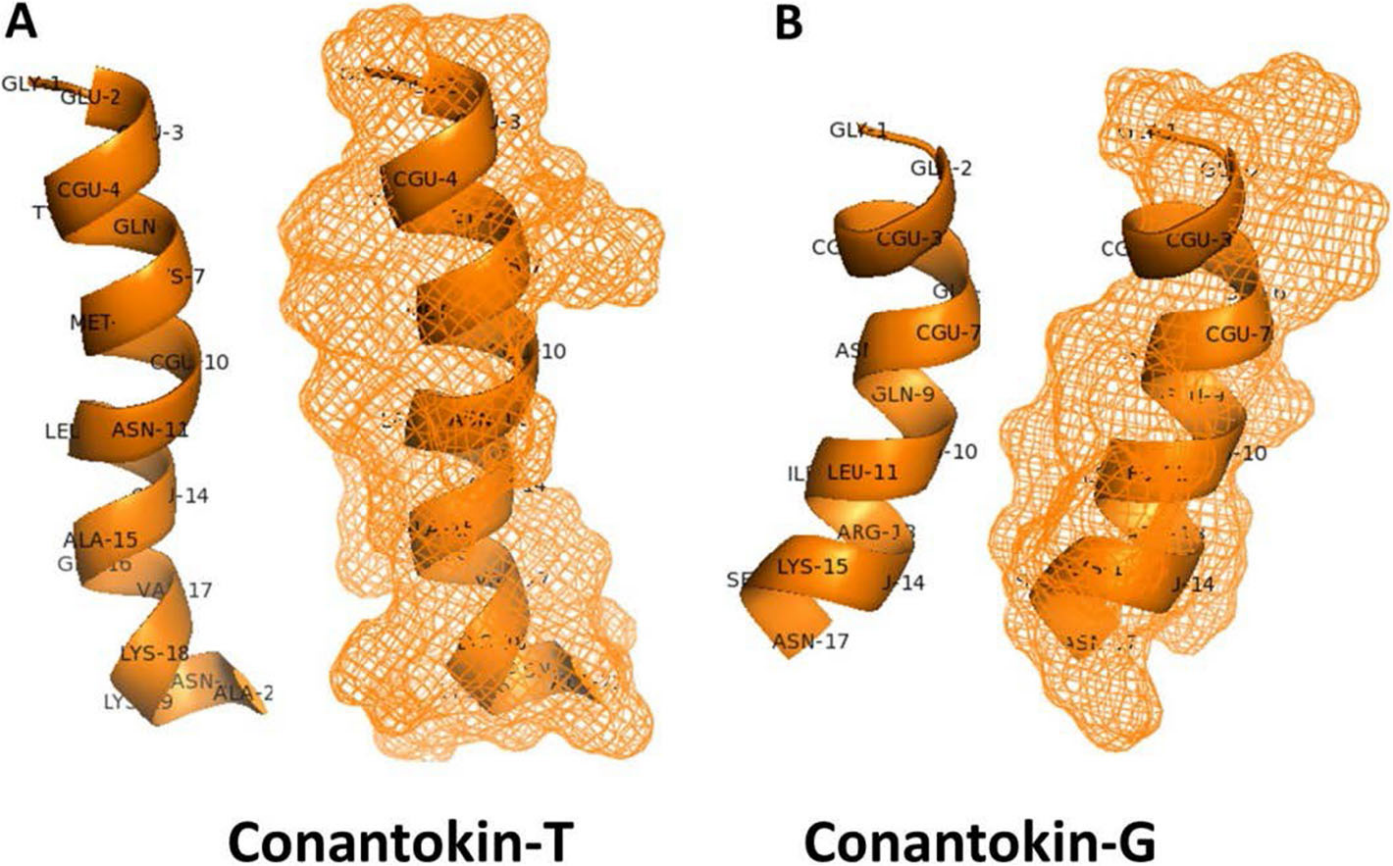
Three-dimensional representation of (**a**) Con-T and (**b**) Con-G, indicating amino acids (*left*) and mesh structure (*right*). CGU = Gla: γ-carboxyglutamic acid. Conantokins adopt an α-helical solution structure in the presence of divalent cations in which charged carboxyl groups of the Gla residues are positioned along one face of the helix and hydrophobic residues are on the opposite face [83]. Figures were created with Pymol [82] (PDB 1ONT and 1ONU for Con-T and Con-G, respectively)

Conantokins show a disproportionately large number of acid labile post-translational γ-carboxyglutamic acid (Gla) residues. These Gla residues play a critical role in the chelation of divalent cations, which causes a stabilization of the α-helical secondary structure of the conantokins [27]. As such, conantokin-G requires the presence of divalent cations to form a stable α-helix, while conantokin-T adopts a stable structure, both in the presence or absence of divalent cations. However, binding of divalent cations increases the α-helical content of both peptides [27]. Moreover, the first five amino acids of conantokins, especially Glu^2^ and Gla^4^ and a hydrophobic residue at position 12 are critical for functional activity in vitro. Position 5 appears to be important in determining the NMDA receptor subtype selectivity profile of the conantokins [27]. For Con-G and Con-T, in particular, Gla^3^ and Gla^4^ have shown to play an important role in the activity of these peptides [32]. Figure 3 shows the three-dimensional structures of Con-T and Con-G.

### NMDA receptors and pharmacological properties of conantokins

The NMDA receptor is a heterooligomeric ligand-gated ion channel mediating excitatory synaptic transmission. The channel has multiple allosterically coupled recognition sites for different components such as glutamate, glycine, polyamines, ions and use-dependent channel blockers. It is a specific type of ionotropic glutamate receptor, because the agonist molecule NMDA (N-methyl-D-aspartate) binds selectively to this receptor and not to other glutamate receptors. At resting membrane potential, NMDA receptors are blocked by Mg^2+^. The activation of NMDA receptors depends on two actions: binding of agonist (such as NMDA) and relief of voltage-dependent channel blockade by Mg^2+^, which happens upon postsynaptic depolarization. As a consequence, NMDA receptors become highly permeable to Ca^2+^ which can influence gene activation, neuronal survival, axon outgrowth and synaptic strength [33]. Native NMDA receptors are composed of subunits from three families, NR1, NR2 and NR3. A single gene encodes the NR1 family, which consists of eight known splice variants. Four different genes encode the members of the NR2 family (NR2A, NR2B, NR2C and NR2D). This latter family is shown to be the target of most conantokins identified up to now.

Conantokins also interact with NMDA receptors of distinct subunit composition. As such, conantokin-G from *Conus geographus* potently inhibits NMDA-evoked currents in *Xenopus* oocytes expressing NR1/NR2B heteromeric receptors [34]. In contrast, conantokin-R potently blocks NMDA-evoked currents in *Xenopus* oocytes expressing NR1/NR2A and NR1/NR2B heteromeric receptors [35].

### Biological role in the venom

The question rises why cone snails have a peptide that functions as a NMDA or Glu antagonist in their venom? When Con-G is injected intramuscularly in fish, a jerky and uncoordinated swimming occurs, which may slow down the fish’s escape. As an alternative, the peptide may act together with other conopeptides to aid in the hunting strategy of the snail. As such, conantokin-G is a component of the nirvana cabal, a synergistic effect caused by different conotoxins, which brings the prey in a relaxed and sedated state. Contulakin-G assists in this nirvana cabal. Another possibility is that some components of the venom are reserved for defensive processes. Since Glu is a neurotransmitter at the invertebrate neuromuscular junction, injection of a potent glutamate antagonist into an invertebrate predator causing the inhibition of signal transmission, may be a very effective defense strategy [30].

### Clinical potential of conantokins

Over-activation of NMDA receptors has been implicated in a variety of neurological disorders including chronic pain, epilepsy, ischemic stroke and neurodegenerative disorders such as Parkinson’s disease and Alzheimer’s disease. Clinical and preclinical studies have demonstrated that antagonists of NMDA receptors may be useful in treating these neurological disorders. Unfortunately, the side effect profiles of many NMDA antagonists (which include psychotomimetic, amnesic and motor-impairing actions and neurotoxicity) limit their usefulness in humans [36, 37].

Conantokins, however, represent a class of NMDA antagonists with an improved safety profile as the following examples illustrate [27]. First, intrathecal delivery of conantokins produces a potent antiallodynic effect in models of tissue or nerve injury induced pain at doses well below those associated with motor impairment. Moreover, it was observed that this antinociceptive effect of conantokin-G only affects the component of pain behavior that involves alterations of nociceptive processing secondary to persistent injury and central sensitization. In contrast, at doses that did not interfere with motor function, conantokins had no effect on “acute” nociceptive responses produced by thermal or chemical stimulation. Therefore, the analgesic properties of conantokins are superior in relation to non-subtype selective NMDA antagonists. Second, conantokins display a broad anticonvulsant profile in experimental seizure models with a large separation between effective and toxic doses in comparison with other classes of NMDA antagonist drugs. Finally, conantokins may represent an important therapeutic tool in the treatment of ischemic stroke, and should theoretically be effective in hemorrhagic stroke where current “clot-busting” drugs are contraindicated.

Con-G and Con-T appear equipotent in antinociceptive and anticonvulsant tests, but Con-G is effective in anti-parkinsonian models while Con-T is not. Nevertheless, the potential pharmacological selectivity of conantokins, coupled with their in vivo efficacy and favorable safety profiles, indicates that these peptides represent an important class of compounds for further evaluation as human therapeutics. As such, CGX-1007, the synthetic variant of conantokin-G, is going through clinical test phases to treat epilepsy [38] (also reviewed in [27, 39–41]). A disadvantage of conantokins is their poor bioavailability, which would require their direct delivery into the central nervous system (intrathecally for instance) [27].

## Contulakin

### General features of contulakin-G

Contulakin-G was discovered in the venom gland of the fish hunting cone snail *Conus geographus* by Craig et al. [18]. An interesting feature of contulakin-G is the glycosylated Thr^10^ (Table 6). Moreover, the C-terminal sequence of contulakin-G shares similarity with an endogenous neurotensin found in vertebrate animals [42]. Consequently, both glycosylated and non-glycosylated analogs of contulakin-G revealed to be potent agonists of rat neurotensin receptor type 1. However, the glycosylated analog is ten-fold more active than the non-glycosylated form. The role of the glycan was first assumed to increase the stability of contulakin-G or to facilitate its transport to the site of action [18]. Later, Lee et al. (2015) found that both glycosylation and charged amino acid residues contribute to the neurotensin receptor desensitization properties of contulakin-G and neurotensin [42].

**Table 6:** Characteristics of contulakin-G. Its amino acid sequence, respective species, target, clinical potential and reference are indicated

Peptide	Amino acid sequence	Species	Target	Clinical potential	Source
Contulakin-G	*Z*SEEGGSNAT^#^KKPYIL	*C. geographus*	Neurotensinreceptor	Pain	[18]

*Z = p*E: pyroglutamate; T^#^: [β-D-Gal-(1 → 3)-α-D-GalNAc-(1→)]Thr

### Neurotensin receptors and pharmacological properties of contulakin-G

Three neurotensin receptors have been identified so far (NTSR1-3), from which both NTSR1 and NTSR2 are G-protein-coupled receptors. Neurotensin (NTS, amino acid sequence ZLYENLPRRPYIL) is a 13-amino-acid peptide that functions both as neurotransmitter and hormone through the activation of NTSR1. This peptide modulates the activity of dopaminergic systems in the brain, but also modulates opioid-independent analgesia and the inhibition of food intake, whereas in the gut, NTS regulates a range of digestive processes [43]. Neurotensin receptors have been studied as molecular targets for the treatment of pain, schizophrenia, addiction or cancer [27]. White et al. [43] determined the crystal structure of *Rattus norvegicus* NTSR1, providing a modeling tool for drug discovery. Recently, Lee et al. [42] used this model in the context of investigating the desensitization properties of both neurotensin and contulakin-G. Compared to neurotensin, contulakin-G is a weaker agonist exhibiting significantly lower desensitization potency. As such, these structure-activity relationship and computer modeling studies suggest that replacements of the charged and glycoamino acid residues in contulakin-G may lead to systemically active NTS analogs with diverse potencies for activating and desensitizing neurotensin receptors [42].

### Biological role in the venom

Looking at the role that contulakin-G might have in capturing fish by *C. geographus*, the peptide is believed to suppress the sensory circuitry in the prey. As *C. geographus* is a net hunter, contulakin-G may be part of eliciting a sedated state in the fish prey [44].

### Clinical potential of contulakin-G

Contulakin-G is a potent analgesic in a number of pain models such as tail-flick (acute pain), formalin test, and CFA-induced allodynia inflammatory pain [18]. Moreover, its synthetic analog, named CGX-1160, reached a clinical development stage for the treatment of chronic intractable pain following intrathecal administration in patients with spinal cord injury [42] (also reviewed in [27, 39–41]). However, when epidural delivery was performed, CGX-1160 was considerably less potent suggesting its restricted ability to diffuse through the meningeal barrier [44].

## Conorfamides

### General features of conorfamides

The first conorfamide, CNF-Sr1 from *C. spurius*, was characterized by Maillo et al. [45] (Table 7). Conorfamide-Sr1 belongs to the RFamide neuropeptide family and was the first RFamide peptide to be found in any venom. The peptide was shown to cause hyperactivity in mice older than 16 days. Another FMRFamide-related peptide (FaRP) is conorfamide-Sr2, described by Aguilar et al. [46]. CNF-Sr2 also produces hyperactivity when injected intracranially into mice and demonstrated other symptoms such as paralysis in snails and limpet [46]. A third conorfamide, Vc1 from *C. victoriae,* showed bioactivity upon injection in mice and calcium imaging of mouse dorsal root ganglion cells revealed that the peptide elicits an increase in intracellular calcium levels [47].

**Table 7:** Characteristics of conorfamides. Their amino acid sequences, respective species, targets and references are indicated

Peptide	Amino acid sequence	Species	Target	Source
Conorfamide-Sr1	GPMGWVPVFYRF^*^	*C. spurius*	RFamide receptor?	[45]
Conorfamide-Sr2	GPMγDPMγIIRI^*^	*C. spurius*	?	[46]
Conorfamide-Vc1	HSGFLLAWSGPRNRFVRF^*^	*C. victoriae*	?	[47]

^*^C-terminal amidation

### RFamide receptors and pharmacological properties of conorfamides

The prototypical RFamide neuropeptide is the tetrapeptide Phe–Met–Arg–Phe–NH_2_ (FMRFamide) that was purified from the clam *Macrocallista nimbosa* on account of its cardioexcitatory activity. In general, RFamide peptides represent a family of regulatory peptides that possess the Arg-Phe-NH_2_ motif at their C-terminus. In mammals, seven RFamide peptides have been characterized [48]. The RFamide distribution patterns appear to be limited to specific brain areas where they are involved in essential functions such as cardiovascular regulation, pain modulation, weight control and food consumption [47, 49].

Several G-protein coupled receptors (GPCRs) have now been identified as receptors for the mammalian RFamide peptides. These include the neuropeptide FF receptors 1 and 2 (NPFF_1_R and NPFF_2_R), the pyroglutamylated RFamide peptide receptor (QRFPR), the kisspeptin receptor and the prolactin-releasing peptide receptor (PrRPR). Each of them is implicated in a series of biological events. As such, NPFF_1_R is localized in the human hypothalamus and surrounding areas supporting the idea that RFamide peptides play a role in central coordination of neuroendocrine and autonomic responses in humans [49]. Next, since QRFP, interacting with QRFPR, is expressed in regions of the hypothalamus involved in control of feeding behavior, this peptide and its receptor are involved in the increasing intake of a high fat diet. Other members of the RFamide family may play roles in the reproductive tract (kisspeptin receptor), or regulate hormone secretion (PrRPR) [50]. Up to now, the specific receptor(s) of the described conorfamides have not yet been discovered.

### Biological role in the venom

In invertebrate nervous systems, the peptides of the RFamide family have a host of diverse functions such as modulation of muscle contraction, control of locomotor activity, regulation of water balance, cardioexcitatory activities and neuromodulatory activities. The RFamide motif appears to be both an ancient and a convergent feature of neuropeptide evolutions [51].

## Conophans

### General features of conophans

Pisarewicz et al. [52] presented four interesting disulfide-poor conopeptides not only incorporating *D*-amino acids into their sequences, but also hydroxyvaline. The peptides containing *D*-Val were termed conophans while the peptides with γ-hydroxyvaline were named γ-hydroxyconophans (Table 8). Conophans are not the only conopeptides including *D*-amino acids, other examples are contryphan-R [20], conomap-Vt [53] and conomarphin [54]. The latter two will be discussed in the following sections. contryphans were discussed earlier.

**Table 8:** Characteristics of conophans. Their amino acid sequences, respective species and references are indicated

Peptide	Amino acid sequence	Species	Target	Source
Conophan gld-V γ–hydroxyconophan gld-V*	AOANSVWS AOANSV*WS	*C. gladiator*	?	[52]
Conophan mus-V γ–hydroxyconophan mus-V*	SOANSVWS SOANSV*WS	*C. mus*	?	[52]

O hydroxyproline; V: *D*-Valine; V*: *D*- γ-hydroxyvaline

The modification of polypeptide chains by epimerization of standard *L*-amino acids to produce their *D*-counterparts is rarely observed [52]. Nevertheless, the modification of *L*- to *D*-amino acids in the spider venom component ω-agatoxin IVB showed to increase stability and potency. As such, *D*-Ser^46^ of ω-agatoxin IVB provides more resistance to the major venom protease and is a more potent blocker of the P-type voltage-sensitive calcium channels than its *L*-ser^46^ counterpart [55]. The observation of a γ-hydroxylation of non-Pro amino acids is even rarer than epimerization. The reason why this event is uncommon is that a hydroxyl group in the γ-position of any amino acid (except Pro) could undergo nucleophilic attack to form a stable five-membered lactone ring. Therefore, the presence of γ-*D*-hydroxyvaline is unexpected. It is hypothesized that the structural motif found in γ-hydroxyconophans, Ser-*D*-Hyv-Trp, is stabilized by specific interactions between the *D*-amino acid and its neighboring *L*-counterparts while these interactions inhibit lactonization [52]. Up to now, the target of conophans remains to be revealed.

## Conomap

### General features of conomap-Vt

Conomap-Vt (Conp-Vt) is an unusual linear tetradecapeptide isolated from *Conus vitulinus* venom. Its sequence displays significant homology (≤ 65 %) to peptides of the MATP (myoactive tetradecapeptide) family, which are important endogenous neuromodulators in mollusks, annelids and insects. Conomap-Vt contains a *D*-phenylalanine at position 2 that was suggested to have evolved for prey capture, since isomerization of *L*-Phe enhanced biological activity of this conopeptide (Table 9) [53].

**Table 9:** Characteristics of conomap-Vt. Its amino acid sequence, respective species, target and reference are indicated

Peptide	Amino acid sequence	Species	Target	Source
Conomap-Vt	AFVKGSAQRVAHGY^a^	*C. vitulinus*	? [excitatory peptide]	[53]

^a^N-terminal amidation; F D-type phenylalanine

### Pharmacological properties of conomap-Vt

Since peptides belonging to the MATP family cause excitatory effects [56, 57], Conp-Vt was tested for excitatory activity in several mollusk isolated tissue preparations such as buccal mass, crop, penis retractor muscle and foot [53]. The initial concentration of Conp-Vt (10 nM) produced a contraction or enhanced existing spontaneous contractions in all tissues except the foot. Further investigations indicated that Conp-Vt has a potent excitatory effect, particularly on the buccal mass that was showed by its EC_50_ of 8 nM [53]. Moreover, Conp-Vt was tested for its ability to induce tachyphylaxis, which is described as the acute loss of response upon repeated stimulations with agonists [58]. Similar to acetylcholine (ACh), repeated doses of Conp-Vt were tachyphylactic. However, it appears that Conp-Vt contractions were non-cholinergic in origin. Finally, the authors concluded that Conp-Vt acts at non-cholinergic receptors to cause the release of ACh and contraction [53].

### Biological role in the venom

Conp-Vt is a major component of the venom of *C. vitulinus*. Therefore, it is suggested that this peptide may play an important role in prey capture. Indeed, a peptide such as Conp-Vt might disrupt muscle contraction, which is an efficient weapon in prey subjugation and capture. However, the function of Conp-Vt is limited to skeletal musculature, since cardiac muscle response was not affected in vertebrates. The authors propose that Conp-Vt has evolved from the cone snail’s own endogenous MATP peptide for specialized venom use [53].

## Conomarphins

### General features of conomarphin

Conomarphin was discovered in 2008 by Han et al. [54]. The cDNA-encoded precursor of conomarphin shares the conserved signal peptide with peptides from the M-superfamily, but the amino acid sequence of this peptide totally differs from other members of this superfamily. Conomarphin is namely a cysteine-free and *D*-amino acid containing conotoxin purified from the venom of *Conus marmoreus*. Moreover, it has a proline residue at position 8, but a hydroxylated proline at position 10 (Table 10) [54].

**Table 10:** Characteristics of conomarphins. Their amino acid sequences, respective species and references are indicated

Peptide	Amino acid sequence	Species	Target	Source
Conomarphin	DWEYHAHPKONSFWT	*C. marmoreus*	?	[54]
Conomarphin-14	DWEYHAHPKONSFW	*C. marmoreus*	?	[60]
Conomarphin-8	HPKONSFW	*C. marmoreus*	?	[60]

O: hydroxyproline; F: *D*-type phenylalanine

**Fig. 4:**
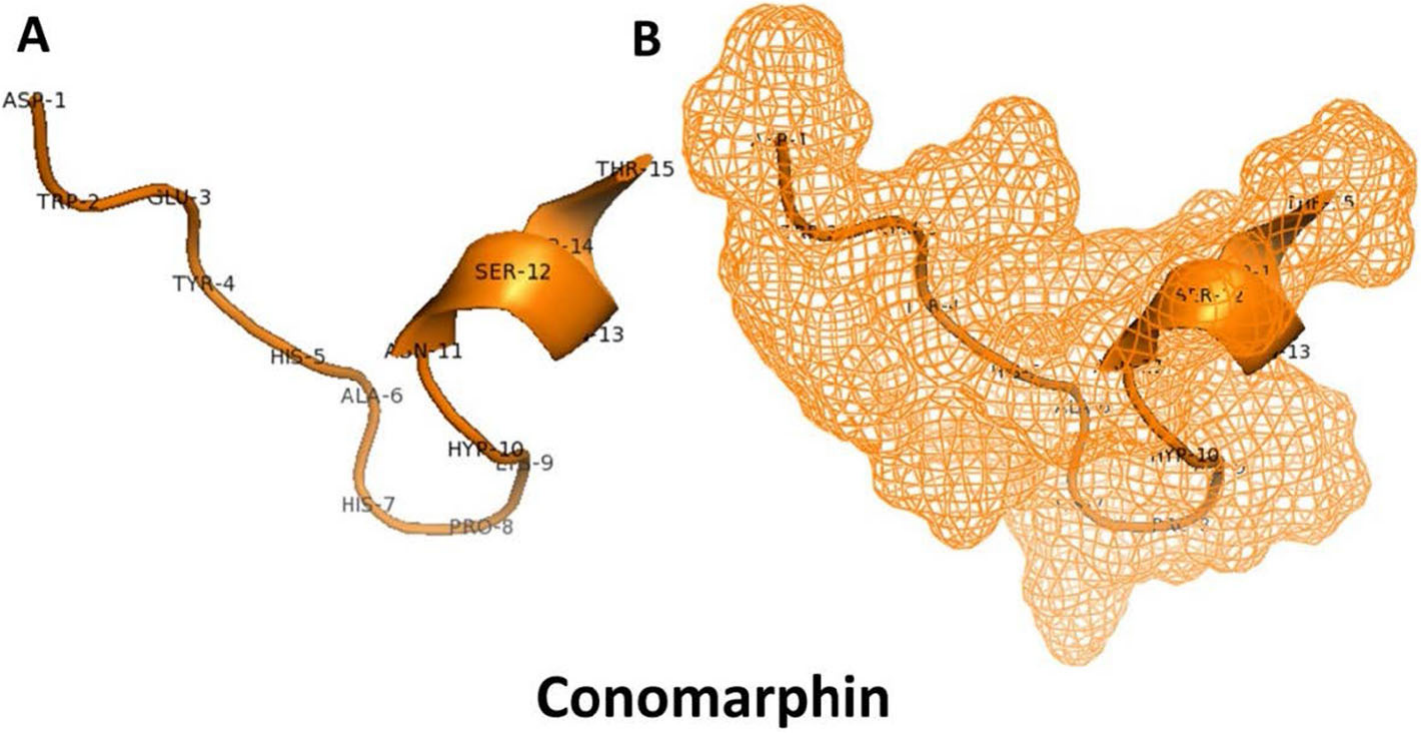
Three-dimensional representation of conomarphin indicating amino acids (a) and mesh structure (b). Figures were created with Pymol [82] (PDB 2YYF)

The three-dimensional structure of conomarphin displays a 3^10^ helix at the C-terminus and a Pro^8^-centered compact loop region (Fig. 4). The authors suggested that *D*-Phe^13^ is essential for the structure of conomarphin, since no loop could be found in *L*-Phe^13^-conomarphin.

Huang and Du [59] investigated the influence of the hydroxyproline at position 10 of conomarphin. This proline hydroxylation occurs with high specificity, since only one of both proline residues is hydroxylated. The authors found that in a Hyp10Pro variant, a type-II β-turn is formed between residues 11 and 14 instead of a 3^10^ helix at the C-terminus of native conomarphin. Thus, the lack of a hydroxyl group changes the electric charge distribution and induces the loss of a hydrogen bond between Pro^10^ and *D*-Phe. This might not only have an impact on the conformation of conomarphin, but also the physiological function [59].

Conomarphin-14 and conomarphin-8 are two variants of conomarphin that were extracted from the venom of *C. marmoreus* by thiol-exchange chromatography and RP-HPLC. Compared to conomarphin, conomarphin-14 misses the C-terminal Thr^15^ residue and in conomarphin-8, a cleavage between Ala^6^ and His^7^ of conomarphin seems to have occurred. Zhang et al. [60] suggested that both conomarphin-8 and conomarphin-14 might have similar structures, because the removed parts are not involved in the formation of conomarphin’s structural elements (3^10^ helix and centered loop). Conomarphin-8 shares significant sequence similarity with the conophans, which implies that the identification of conomarphin-8 is not an artifact. Consequently, the authors raise some intriguing questions: “Is it possible that these short peptides like conomarphin-8 and conophans are actually the final products, and that conomarphin and conomarphin-14 are only intermediates of the maturation process of conomarphin-8? Or do both conomarphin-8 and conomarphin have their separate biological functions?” These points certainly need further investigation.

## Conolysins

### General features of conolysins

Conolysins are conopeptides that have the ability to disrupt the integrity of cell membranes. The first conopeptide shown to have this cytolytic activity was conolysin-Mt from *Conus mustelinus* (Table 11). Cytolytic peptides are among the largest group of toxins produced by living organisms, which include bacteria, viruses, insects, marine invertebrates and reptiles [61].

**Table 11:** Characteristics of conolysins. Their amino acid sequences, respective species, targets and references are indicated

Peptide	Amino acid sequence	Species	Target	Source
Conolysin-Mt1	FHPSLWVLIPQYIQLIRKILKSG	*C. mustelinus*	cellular membranes	[61]
Conolysin-Mt2	FHPSLWVLIPQYIQLIRKILKS^a^	*C. mustelinus*	cellular membranes	[61]

^a^C-terminal amidation

### Pharmacological properties of conolysins

When conolysin-Mt is intracranially injected into 16-day-old Swiss-Webster mice, a backward shuffling, like a moonwalk, is observed. When the mouse touches an obstacle during a moonwalk, it jumps in the air and often goes into a seizure. Therefore, these peptides are named “moonwalker peptides”. Both conolysin-Mt1 and Mt2 were found in the venom of *C. mustelinus*. The least abundant of the two peptides (Mt1) was more active in this moonwalker assay, causing the full moonwalker phenotype to be observed within 35 min using roughly 3 nmol. Mt2 elicited the phenotype in 150 min, using roughly 12 nmol. To reveal the peptides’ target, synthetic Mt1 was tested on *Xenopus* oocytes showing a collapsed membrane potential within seconds. Within minutes, visible holes in the oocytes were observed, revealing this toxin as a cytolytic toxin. Moreover, the peptide was tested against three bacterial strains: *E. coli* D21, *E. coli* ATCC 25922, and *S. aureus* ATCC 6538. It exhibited low antibacterial activity against the two *E. coli* strains tested, with the minimal inhibitory concentration (MIC) > 50 μM. The MIC against the gram-positive *S. aureus* was 25–50 μM. To explain the low antibacterial activity of conolysin-Mt, it was suggested that larger aggregates might fail to pass the outer barrier of bacteria. In agreement, the lower MIC-values for *S. aureus* suggest that the peptide is more active against gram-positive rather than gram-negative bacteria, as the latter has an additional outer membrane. To put things in perspective, melittin – the main peptide in *Apis melifera* (honey bee) venom – exhibits potent antibacterial activity on both gram-negative and gram-positive bacteria. Its antibacterial activity under the same assay condition is 1–20 μM, depending on the bacterial strain used [61].

## Cono-NPYs

### General features of cono-NPYs

Wu et al. [62] described two neuropeptide Y (NPY)-like exocrine conopeptides, identified in the venom of *Conus betulinus*. NPY (YPSKPDNPGEDAPAEDLARYYSALRHYINLITRQRY^a^ with a: N-terminal amidation) is an endocrine neurotransmitter found in the brain and autonomic nervous system of both vertebrates and invertebrates. It is associated with a number of biological functions, such as feeding behavior, energy balance, blood pressure, circadian rhythms, sexual behavior, memory processing, cognition, anxiety and epilepsy [63–65] Several NPY receptors have been identified in mammals and all of them are G-protein coupled receptors (GPCRs).

### Pharmacological properties of cono-NPYs

The bioactivities of cono-NPYs (Table 12) were assayed by intraventricular injection of these peptides into mice brain. At a dose of 20 μg/mouse, the mice showed signs of hyperactivity, such as jumping, rapid circling, and tail flicking. The symptoms appeared 20 s after injection and lasted for 40–60 s. Then, the mice calmed down and recovered. Despite these biological activities, it remains to be clarified if their real receptors are NPY receptors or related GPCRs [62].

**Table 12:** Characteristics of cono-NPYs. Their amino acid sequences, respective species and references are indicated

Peptide	Amino acid sequence	Species	Target	Source
Cono-NPY	TVSDPPARPAVFHSREELMNYVRELNRYFAIVGRPRY^a^	*C. betulinus*	?	[62]
Cono-NPF	TVSDPPARPAVFHSREELMNYVRELNRYFAIVGRPRF^a^	*C. betulinus*	?	[66]

^a^N-terminal amidation

## Other hormone-like conopeptides

Recently, two new hormone-like conopeptides, elevenin-Vc1 and PH4-Vc1 (prohormone-4), were described by Robinson et al. [66] (Table 13). *C. victoriae* elevenin was identified as a 19 amino acid mature peptide with one pair of cysteines. Other elevenin transcripts have been identified in gastropod mollusks and annelids. Prohormone-4 precursors were identified in the venom gland transcriptomes of several other species of *Conus* (*C. geographus, C. tessulatus, C. varius* and *C. virgo*). In honey bee, prohormone-4 was identified in the brain and has been implicated in the regulation of behavior [66].

**Table 13:** Characteristics of two hormone-like conopeptides. Their amino acid sequences, respective species and references are indicated

Peptide	Amino acid sequence	Species	Target	Source
Elevenin-Vc1	RRIDCKVFVFAPICRGVAA	*C. victoriae*	?	[66]
Prohormone-4-Vc1	IGFPGFSTPPR	*C. victoriae*	?	[66]

Both conopeptides are part of the injected venom cocktail of *C. victoriae*, unambiguously demonstrating their role in envenomation. These and several other previously reported venom components (insulin, thyrostimulin, conopressin, conoCAPs, conomap) seem to be recruited into the venom from endogenous processes. Up to now, the molecular targets of both elevenin and prohormone-4 remain to be determined.

## Conclusion

*Conus* venoms contain a remarkable diversity of pharmacologically active small peptides. Of this venom cocktail, disulfide-poor conotoxins are mostly minor components. Moreover, an even smaller portion has only one disulfide bond, which makes cysteine-poor conopeptides less investigated than its cysteine-rich equivalents. This review provides an overview of 12 families of disulfide-poor peptides. As such, a description of the chemical features, pharmacological properties and the biological role of the venom components is given. Nevertheless, numerous questions still need to be answered. These include the determination of specific targets or mechanisms of action and their biological role in the venom that still remains to be elucidated and, on the other hand, the lack of structure-activity data.

From the limited number of disulfide-poor conopeptides characterized up to now, two (contulakin-G and conantokin-G) have already reached clinical trials, which indicates *Conus* venoms as promising compounds for drug discovery. Since disulfide-poor conotoxins do not suffer from folding problems encountered with multiple disulfide bridges, they are interesting scaffolds for drug discovery. Moreover, cyclic structures – such as conopressin, contryphans and conoGAYs – are remarkable compounds when it comes to bioavailability. It has namely been proven that cyclic structures are more stable and less sensitive to proteolysis [67]. Therefore, we are hopeful that these peptides will get more attention in future research. A final overview of the different families of disulfide-poor conopeptides is given in Table 14.

**Table 14:** Overview of the different families of disulfide-poor conopeptides discussed in this review. The different conopeptide families, a representative peptide and amino acid sequence of this peptide, respective *Conus* species, targets and, when applicable, clinical applications are indicated

Family	Peptide representative	Sequence	*Conus* species	Target	Clinical applications
Conopressin	Lys-conopressin-G	CFIRNCPKG^a^	*C. geographus*	Vasopressin receptor	Cardiovascular/mood
Contryphan	Contryphan-R	GCOWEPWC^a^	*C. radiatus*	VGPC, VGCC	
ConoCAP	ConoCAP-a	PFCNSFGCYN^a^	*C. villipinii*	?	
ConoGAY	ConoGAY-AusB	GAYFDGFDVPCVPRRDDC	*C. australis*	?	
Conantokin	Con-G	GEγγLQγNQγLIRγKSN^a^	*C. geographus*	NMDA receptor	Pain/epilepsy
Contulakin	Cont-G	ZSEEGGSNAT^#^KKPYIL	*C. geographus*	Neurotensin Receptor	Pain
Conorfamides	Conorfamide-Sr1	GPMGWVPVFYRF^a^	*C. spurius*	RFamide receptor?	
Conophan	Conophan Gld-V	AOANSVWS	*C. gladiator*	?	
Conomaps	Conp-Vt	AFVKGSAQRVAHGY^a^	*C. vitelinus*	?[Excitatory peptide]	
Conomarphin	Conomarphin	DWEYHAHPKONSFWT	*C. marmoreus*	?	
Conolysin	Conolysin-Mt2	FHPSLWVLIPQYIQLIRKILKS^a^	*C. mustelinus*	Cellular membranes	
Cono-NPY	Cono-NPY	TVSDPPARPAVFHSREELMNYVRELNRYFAIVGRPRY^a^	*C. betulinus*	?	
Hormone-like conopeptides	Elevenin PH-4	RRIDCKVFVFAPICRGVAA IGFPGFSTPPR	*C. victoriae*	?	

^a^N-terminal amidation; W: $D$ -tryptophan; γ = Gla: γ-carboxyglutamic acid; Z = pE: pyroglutamaat; T^#^: Gal-GalNAc-Thr; V: $D$ -Valine; F: $D$ -Phe

## Abbreviations

AVP: Vasopressin
AVT: Vasotocin
Ca_V_ = VGCC: Voltage-gated calcium channels
CFA: Complete Freund’s adjuvant
CNF: Conorfamide
Con: Conantokin
Conp: Conomap
Cont: Contulakin
FaRP: FMRFamide-related peptide
Gla: γ-carboxyglutamic acid
GPCR: G-protein-coupled receptor
HVA: High-voltage activated
K_V_ = VGPC: Voltage-gated potassium channels
LTTC: L-type Ca^2+^ channel
MATP: Myoactive tetradecapeptide
MIC: Minimal inhibitory concentration
NMDA: N-methyl-D aspartate
NPFFR: Neuropeptide FF receptor
NPY: Neuropeptide-Y
NTS: Neurotensin
OT(R): Oxytocin (receptor)
VR: Vasopressin receptor

## References

[1] Norton RS, Olivera BM (2006). Conotoxins down under. Toxicon.

[2] Davis J, Jones A, Lewis RJ (2009). Remarkable inter- and intra-species complexity of conotoxins revealed by LC/MS. Peptides.

[3] Puillandre N, Koua D, Favreau P, Olivera BM, Stöcklin R (2012). Molecular phylogeny, classification and evolution of conopeptides. J Mol Evol.

[4] Olivera BM, Rivier J, Clark C, Ramilo CA, Corpuz GP, Abogadie FC (1990). Diversity of conus neuropeptides. Science.

[5] Craik DJ, Adams DJ (2007). Chemical modification of conotoxins to improve stability and activity. ACS Chem Biol.

[6] Olivera BM, Teichert RW (2007). Diversity of the neurotoxic conus peptides: a model for concerted pharmacological discovery. Mol Interv.

[7] Akondi KB, Muttenthaler M, Dutertre S, Kaas Q, Craik DJ, Lewis RJ (2014). Discovery, synthesis, and structure-activity relationships of conotoxins. Chem Rev.

[8] Cruz LJ, de Santos V, Zafaralla GC, Ramilo CA, Zeikus R, Gray WR (1987). Invertebrate vasopressin/oxytocin homologs. Characterization of peptides from conus geographus and conus straitus venoms. J Biol Chem.

[9] Nielsen DB, Dykert J, Rivier JE, McIntosh JM (1994). Isolation of Lys-conopressin-G from the venom of the worm-hunting snail, *conus imperialis*. Toxicon.

[10] McMaster D, Kobayashi Y, Lederis K (1992). A vasotocin-like peptide in aplysia kurodai ganglia: HPLC and RIA evidence for its identity with Lys-conopressin G. Peptides.

[11] Salzet M, Bulet P, Van Dorsselaer A, Malecha J (1993). Isolation, structural characterization and biological function of a lysine-conopressin in the central nervous system of the pharyngobdellid leech erpobdella octoculata. Eur J Biochem.

[12] Van Kesteren RE, Smit AB, De Lange RP, Kits KS, Van Golen FA, Van Der Schors RC (1995). Structural and functional evolution of the vasopressin/oxytocin superfamily: vasopressin-related conopressin is the only member present in Lymnaea, and is involved in the control of sexual behavior. J Neurosci.

[13] Moller C, Mari F (2007). A vasopressin/oxytocin-related conopeptide with gamma-carboxyglutamate at position 8. Biochem J.

[14] Banerjee P, Joy KP, Chaube R. Structural and functional diversity of nonapeptide hormones from an evolutionary perspective: A review. Gen Comp Endocrinol. 2016;(16):30107-11. doi:10.1016/j.ygcen.2016.04.025. 10.1016/j.ygcen.2016.04.025 27133544

[15] McCormick SD, Bradshaw D (2006). Hormonal control of salt and water balance in vertebrates. Gen Comp Endocrinol.

[16] Veenema AH, Blume A, Niederle D, Buwalda B, Neumann ID (2006). Effects of early life stress on adult male aggression and hypothalamic vasopressin and serotonin. Eur J Neurosci.

[17] Dutertre S, Croker D, Daly NL, Andersson A, Muttenthaler M, Lumsden NG (2008). Conopressin-T from Conus tulipa reveals an antagonist switch in vasopressin-like peptides. J Biol Chem.

[18] Craig AG, Norberg T, Griffin D, Hoeger C, Akhtar M, Schmidt K (1999). Contulakin-G, an O-glycosylated invertebrate neurotensin. J Biol Chem.

[19] van Kesteren RE, Smit AB, Dirks RW, de With ND, Geraerts WP, Joosse J (1992). Evolution of the vasopressin/oxytocin superfamily: characterization of a cDNA encoding a vasopressin-related precursor, preproconopressin, from the mollusc *Lymnaea stagnalis*. Proc Natl Acad Sci U S A.

[20] Jimenéz EC, Olivera BM, Gray WR, Cruz LJ (1996). Contryphan is a D-tryptophan-containing conus peptide. J Biol Chem.

[21] Jimenez EC, Craig AG, Watkins M, Hillyard DR, Gray WR, Gulyas J (1997). Bromocontryphan: post-translational bromination of tryptophan. Biochemistry.

[22] Grant MA, Hansson K, Furie BC, Furie B, Stenflo J, Rigby AC (2004). The metal-free and calcium-bound structures of a gamma-carboxyglutamic acid-containing contryphan from conus marmoreus, glacontryphan-M. J Biol Chem.

[23] Pallaghy PK, Melnikova AP, Jimenez EC, Olivera BM, Norton RS (1999). Solution structure of contryphan-R, a naturally occurring disulfide-bridged octapeptide containing D-tryptophan: comparison with protein loops. Biochemistry.

[24] Massilia GR, Eliseo T, Grolleau F, Lapied B, Barbier J, Bournaud R (2003). Contryphan-Vn: a modulator of Ca2 + -dependent K+ channels. Biochem Biophys Res Commun.

[25] Sabareesh V, Gowd KH, Ramasamy P, Sudarslal S, Krishnan KS, Sikdar SK (2006). Characterization of contryphans from *conus loroisii* and *conus amadis* that target calcium channels. Peptides.

[26] Moller C, Melaun C, Castillo C, Diaz ME, Renzelman CM, Estrada O (2010). Functional hypervariability and gene diversity of cardioactive neuropeptides. J Biol Chem.

[27] Layer RT, Wagstaff JD, White HS (2004). Conantokins: peptide antagonists of NMDA receptors. Curr Med Chem.

[28] McIntosh JM, Olivera BM, Cruz LJ, Gray WR (1984). Gamma-carboxyglutamate in a neuroactive toxin. J Biol Chem.

[29] Haack JA, Rivier J, Parks TN, Mena EE, Cruz LJ, Olivera BM (1990). Conantokin-T. A gamma-carboxyglutamate containing peptide with N-methyl-d-aspartate antagonist activity. J Biol Chem.

[30] Mena EE, Gullak MF, Pagnozzi MJ, Richter KE, Rivier J, Cruz LJ (1990). Conantokin-G: a novel peptide antagonist to the N-methyl-D-aspartic acid (NMDA) receptor. Neurosci Lett.

[31] Rivier J, Galyean R, Simon L, Cruz LJ, Olivera BM, Gray WR (1987). Total synthesis and further characterization of the gamma-carboxyglutamate-containing “sleeper” peptide from *conus geographus* venom. Biochemistry.

[32] Zhou LM, Szendrei GI, Fossom LH, Maccecchini ML, Skolnick P, Otvos L (1996). Synthetic analogues of conantokin-G: NMDA antagonists acting through a novel polyamine-coupled site. J Neurochem.

[33] Dingledine R, Borges K, Bowie D, Traynelis SF (1999). The glutamate receptor ion channels. Pharmacol Rev.

[34] Donevan SD, McCabe RT (2000). Conantokin G is an NR2B-selective competitive antagonist of N-methyl-D-aspartate receptors. Mol Pharmacol.

[35] White HS, McCabe RT, Armstrong H, Donevan SD, Cruz LJ, Abogadie FC (2000). In vitro and in vivo characterization of conantokin-R, a selective NMDA receptor antagonist isolated from the venom of the fish-hunting snail *Conus radiatus*. J Pharmacol Exp Ther.

[36] Sveinbjornsdottir S, Sander JW, Upton D, Thompson PJ, Patsalos PN, Hirt D (1993). The excitatory amino acid antagonist D-CPP-ene (SDZ EAA-494) in patients with epilepsy. Epilepsy Res.

[37] Muir KW, Grosset DG, Gamzu E, Lees KR (1994). Pharmacological effects of the non-competitive NMDA antagonist CNS 1102 in normal volunteers. Br J Clin Pharmacol.

[38] Bialer M, Johannessen SI, Kupferberg HJ, Levy RH, Loiseau P, Perucca E (2002). Progress report on new antiepileptic drugs: a summary of the Sixth Eilat Conference (EILAT VI). Epilepsy Res.

[39] Han TS, Teichert RW, Olivera BM, Bulaj G (2008). Conus venoms - a rich source of peptide-based therapeutics. Curr Pharm Des.

[40] Layer RT, McIntosh JM (2006). Conotoxins: therapeutic potential and application. Mar Drugs.

[41] Lewis RJ, Dutertre S, Vetter I, Christie MJ (2012). Conus venom peptide pharmacology. Pharmacol Rev.

[42] Lee HK, Zhang L, Smith MD, Walewska A, Vellore NA, Baron R (2015). A marine analgesic peptide, contulakin-G, and neurotensin are distinct agonists for neurotensin receptors: uncovering structural determinants of desensitization properties. Front Pharmacol.

[43] White JF, Noinaj N, Shibata Y, Love J, Kloss B, Xu F (2012). Structure of the agonist-bound neurotensin receptor. Nature.

[44] Kern SE, Allen J, Wagstaff J, Shafer SL, Yaksh T (2007). The pharmacokinetics of the conopeptide contulakin-G (CGX-1160) after intrathecal administration: an analysis of data from studies in beagles. Anesth Analg.

[45] Maillo M, Aguilar MB, Lopez-Vera E, Craig AG, Bulaj G, Olivera BM (2002). Conorfamide, a conus venom peptide belonging to the RFamide family of neuropeptides. Toxicon.

[46] Aguilar MB, Luna-Ramirez KS, Echeverria D, Falcon A, Olivera BM, de la Cotera EP H (2008). Conorfamide-Sr2, a gamma-carboxyglutamate-containing FMRFamide-related peptide from the venom of conus spurius with activity in mice and mollusks. Peptides.

[47] Robinson SD, Safavi-Hemami H, Raghuraman S, Imperial JS, Papenfuss AT, Teichert RW (2015). Discovery by proteogenomics and characterization of an RF-amide neuropeptide from cone snail venom. J Proteomics.

[48] Chartrel N, Alonzeau J, Alexandre D, Jeandel L, Alvear-Perez R, Leprince J (2011). The RFamide neuropeptide 26RFa and its role in the control of neuroendocrine functions. Front Neuroendocrinol.

[49] Findeisen M, Rathmann D, Beck-Sickinger AG (2011). Structure-activity studies of RFamide peptides reveal subtype-selective activation of neuropeptide FF1 and FF2 receptors. ChemMedChem.

[50] Dockray GJ (2004). The expanding family of RF-amide peptides and their effects on feeding behaviour. Exp Physiol.

[51] Elphick MR, Mirabeau O (2014). The evolution and variety of RFamide-type neuropeptides: insights from deuterostomian invertebrates. Front Endocrinol.

[52] Pisarewicz K, Mora D, Pflueger FC, Fields GB, Mari F (2005). Polypeptide chains containing D-gamma-hydroxyvaline. J Am Chem Soc.

[53] Dutertre S, Lumsden NG, Alewood PF, Lewis RJ (2006). Isolation and characterisation of conomap-Vt, a D-amino acid containing excitatory peptide from the venom of a vermivorous cone snail. FEBS Lett.

[54] Han Y, Huang F, Jiang H, Liu L, Wang Q, Wang Y (2008). Purification and structural characterization of a D-amino acid-containing conopeptide, conomarphin, from conus marmoreus. FEBS J.

[55] Heck SD, Siok CJ, Krapcho KJ, Kelbaugh PR, Thadeio PF, Welch MJ (1994). Functional consequences of posttranslational isomerization of Ser46 in a calcium channel toxin. Science.

[56] Li KW, Holling T, de With ND, Geraerts WP (1993). Purification and characterization of a novel tetradecapeptide that modulates oesophagus motility in *lymnaea stagnalis*. Biochem Biophys Res Commun.

[57] Ukena K, Oumi T, Morishita F, Furukawa Y, Matsushima O, Takahama H (1997). Immunochemical demonstration of eisenia tetradecapeptide, a bioactive peptide isolated from the gut of the earthworm eisenia foetida, in tissues of the earthworm. Cell Tissue Res.

[58] Martinez-Mir I, Gil Marques M, Morales-Olivas FJ, Rubio-Gomis E (2002). Characteristics of histamine tachyphylaxis in rat uterine smooth muscle. Inflamm Res.

[59] Huang F, Du W (2009). Solution structure of Hyp10Pro variant of conomarphin, a cysteine-free and D-amino-acid containing conopeptide. Toxicon.

[60] Zhang L, Shao X, Chi C, Wang C (2010). Two short D-Phe-containing cysteine-free conopeptides from *conus marmoreus*. Peptides.

[61] Biggs JS, Rosenfeld Y, Shai Y, Olivera BM (2007). Conolysin-Mt: a conus peptide that disrupts cellular membranes. Biochemistry.

[62] Wu X, Shao X, Guo ZY, Chi CW (2010). Identification of neuropeptide Y-like conopeptides from the venom of *conus betulinus*. Acta Biochim Biophys Sin Shanghai.

[63] Benarroch EE (2009). Neuropeptide Y: its multiple effects in the CNS and potential clinical significance. Neurology.

[64] Hokfelt T, Stanic D, Sanford SD, Gatlin JC, Nilsson I, Paratcha G (2008). NPY and its involvement in axon guidance, neurogenesis, and feeding. Nutrition.

[65] Brain SD, Cox HM (2006). Neuropeptides and their receptors: innovative science providing novel therapeutic targets. Br J Pharmacol.

[66] Robinson SD, Li Q, Bandyopadhyay PK, Gajewiak J, Yandell M, Papenfuss AT, et al. Hormone-like peptides in the venoms of marine cone snails. Gen Comp Endocrinol. 2015;(15):00214-22. doi:10.1016/j.ygcen.2015.07.012. 10.1016/j.ygcen.2015.07.012 PMC4762756 26301480

[67] Clark RJ, Jensen J, Nevin ST, Callaghan BP, Adams DJ, Craik DJ (2010). The engineering of an orally active conotoxin for the treatment of neuropathic pain. Angew Chem Int Ed Engl.

[68] Ueberheide BM, Fenyo D, Alewood PF, Chait BT (2009). Rapid sensitive analysis of cysteine rich peptide venom components. Proc Natl Acad Sci U S A.

[69] Jacobsen R, Jimenez EC, Grilley M, Watkins M, Hillyard D, Cruz LJ (1998). The contryphans, a D-tryptophan-containing family of conus peptides: interconversion between conformers. J Pept Res.

[70] Jacobsen RB, Jimenez EC, De la Cruz RG, Gray WR, Cruz LJ, Olivera BM (1999). A novel D-leucine-containing conus peptide: diverse conformational dynamics in the contryphan family. J Pept Res.

[71] Jimenez EC, Watkins M, Juszczak LJ, Cruz LJ, Olivera BM (2001). Contryphans from conus textile venom ducts. Toxicon.

[72] Massilia GR, Schinina ME, Ascenzi P, Polticelli F (2001). Contryphan-Vn: a novel peptide from the venom of the Mediterranean snail conus ventricosus. Biochem Biophys Res Commun.

[73] Hansson K, Ma X, Eliasson L, Czerwiec E, Furie B, Furie BC (2004). The first gamma-carboxyglutamic acid-containing contryphan. A selective L-type calcium ion channel blocker isolated from the venom of conus marmoreus. J Biol Chem.

[74] Thakur SS, Balaram P (2007). Rapid mass spectral identification of contryphans. Detection of characteristic peptide ions by fragmentation of intact disulfide-bonded peptides in crude venom. Rapid Commun Mass Spectrom.

[75] Nepravishta R, Mandaliti W, Melino S, Eliseo T, Paci M (2014). Structure of the cyclic peptide [W8S] contryphan Vn: effect of the tryptophan/serine substitution on trans-cis proline isomerization. Amino Acids.

[76] Rajesh RP (2015). Novel M-superfamily and T-superfamily conotoxins and contryphans from the vermivorous snail *conus figulinus*. J Pept Sci.

[77] Gowd KH, Twede V, Watkins M, Krishnan KS, Teichert RW, Bulaj G (2008). Conantokin-P, an unusual conantokin with a long disulfide loop. Toxicon.

[78] Jimenez EC, Donevan S, Walker C, Zhou LM, Nielsen J, Cruz LJ (2002). Conantokin-L, a new NMDA receptor antagonist: determinants for anticonvulsant potency. Epilepsy Res.

[79] Franco A, Pisarewicz K, Moller C, Mora D, Fields GB, Mari F (2006). Hyperhydroxylation: a new strategy for neuronal targeting by venomous marine molluscs. Prog Mol Subcell Biol.

[80] Teichert RW, Jimenez EC, Twede V, Watkins M, Hollmann M, Bulaj G (2007). Novel conantokins from Conus parius venom are specific antagonists of N-methyl-D-aspartate receptors. J Biol Chem.

[81] Twede VD, Teichert RW, Walker CS, Gruszczynski P, Kazmierkiewicz R, Bulaj G (2009). Conantokin-Br from conus brettinghami and selectivity determinants for the NR2D subunit of the NMDA receptor. Biochemistry.

[82] Schrödinger L. The PyMOL Molecular Graphics System, Version 1.8 Schrödinger, LLC. Available online: www.pymol.org. Accessed 20 Aug 2016.

[83] Skjaerbaek N, Nielsen KJ, Lewis RJ, Alewood P, Craik DJ (1997). Determination of the solution structures of conantokin-G and conantokin-T by CD and NMR spectroscopy. J Biol Chem.

[84] Lebbe EKM, Ghequire MGK, Peigneur S, Mille BG, Devi P, Ravichandran S, Waelkens E, D’Souza L, De Mot R, Tytgat J (2016). Novel Conopeptides of Largely Unexplored Indo Pacific Conus sp. Mar. Drugs.

